# Development of Sorghum Genotypes for Improved Yield and Resistance to Grain Mold Using Population Breeding Approach

**DOI:** 10.3389/fpls.2021.687332

**Published:** 2021-07-28

**Authors:** C. Aruna, I. K. Das, P. Sanjana Reddy, R. B. Ghorade, A. R. Gulhane, V. V. Kalpande, S. T. Kajjidoni, N. G. Hanamaratti, S. N. Chattannavar, Shivaji Mehtre, Vikram Gholve, K. R. Kamble, C. Deepika, N. Kannababu, D. M. Bahadure, Mahalingam Govindaraj, V. A. Tonapi

**Affiliations:** ^1^ICAR-Indian Institute of Millets Research, Hyderabad, India; ^2^Dr. Panjabrao Deshmukh Krishi Vidyapeeth, Akola, India; ^3^UAS, Dharwad, India; ^4^Vasantrao Naik Marathwada Krishi Vidyapeeth, Parbhani, India; ^5^International Crops Research Institute for Semi-arid Tropics, Patancheru, India

**Keywords:** grain mold, population breeding, GGE biplot, G×E interactions, glume cover, grain hardness

## Abstract

The infection caused by grain mold in rainy season grown sorghum deteriorates the physical and chemical quality of the grain, which causes a reduction in grain size, blackening, and making them unfit for human consumption. Therefore, the breeding for grain mold resistance has become a necessity. Pedigree breeding has been widely used across the globe to tackle the problem of grain mold. In the present study, a population breeding approach was employed to develop genotypes resistant to grain mold. The complex genotype × environment interactions (GEIs) make the task of identifying stable grain mold-resistant lines with good grain yield (GY) challenging. In this study, the performance of the 33 population breeding derivatives selected from the four-location evaluation of 150 genotypes in 2017 was in turn evaluated over four locations during the rainy season of 2018. The Genotype plus genotype-by-environment interaction (GGE) biplot analysis was used to analyze a significant GEI observed for GY, grain mold resistance, and all other associated traits. For GY, the location explained a higher proportion of variation (51.7%) while genotype (G) × location (L) contributed to 21.9% and the genotype contributed to 11.2% of the total variation. For grain mold resistance, G × L contributed to a higher proportion of variation (30.7%). A graphical biplot approach helped in identifying promising genotypes for GY and grain mold resistance. Among the test locations, Dharwad was an ideal location for both GY and grain mold resistance. The test locations were partitioned into three clusters for GY and two clusters for grain mold resistance through a “which-won-where” study. Best genotypes in each of these clusters were selected. The breeding for a specific cluster is suggested. Genotype-by-trait biplots indicated that GY is influenced by flowering time, 100-grain weight (HGW), and plant height (PH), whereas grain mold resistance is influenced by glume coverage and PH. Because GY and grain mold score were independent of each other, there is a scope to improve both yield and resistance together.

## Introduction

Sorghum is well-adapted to the harsh conditions of arid and semiarid agroecologies where other crops fail to thrive well. Sorghum grain has a wide range of uses such as food, feed, brewing, and grain-based ethanol production (Ashok Kumar et al., 2011; Gonzalez et al., 2011; Aruna et al., 2019). Though sorghum has an important role in different sectors, the availability of good quality grain in an adequate amount is greatly influenced by climate variability, which induces constraints in terms of biotic and abiotic stresses (Das et al., 2012; Tovignan et al., 2016). Changed climate scenario, cropping pattern, and the cultivation of high-yielding hybrids and varieties have brought a change in the disease scenario over time, and the diseases, which were considered minor at some point of time, have become predominant, thereby inducing losses in yield and quality. Grain mold of rainy season grown sorghum is one such disease, which has changed its significance over time (Das et al., 2020). Now, it has become a prominent disease of rainy season sorghum for its devastating effects on grain yield (GY) and quality. It is a common disease in the countries of Asia, Africa, North America, and South America (Frederiksen et al., 1982; Das and Padmaja, 2016). The severity of the disease has increased in Asia and Africa where white grain sorghum is grown for food use. In India, high-yielding white grain hybrids are widely grown during the rainy season, which are devastated due to grain mold. This is mainly due to the cultivation of short- and medium-duration sorghum cultivars that mature during the rainy days in humid, tropical, and subtropical climates. These cultivars become susceptible to the disease while late-maturing photoperiod-sensitive sorghums generally escape grain mold as they attain maturity during dry weather (Rao et al., 2013). The disease has adverse effects on GY, quality, market value, seed quality, and eventually on the end products developed from grain (Das, 2019). Mold attack has become one of the major constraints hindering sorghum cultivation, which resulted in a drastic decrease in the area of cultivation. GY reduction up to 30–100% was reported (Ashok Kumar et al., 2011) with the estimated annual loss of US$ 130 million across the globe (ICRISAT, 1992) and US$ 50–80 million in India (Das and Patil, 2013). Yield losses up to 100% are reported on grain mold-susceptible cultivars under conducive environmental conditions such as rains during grain maturity (Singh and Bandyopadhyay, 2000; Navi et al., 2005). The grain mold causes yield reductions by poor seed formation due to caryopsis abortion, reduced grain filling, and filling grains with less density. The grain quality and thereby the market value is affected due to surface discoloration, poor grain size, and density. In addition, grain mold poses health hazards to both human and animals due to the production of mycotoxins and secondary metabolites (Castor and Frederiksen, 1980; Little and Magill, 2009; Audilakshmi et al., 2011).

The prevailing weather conditions, particularly high relative humidity (RH) and temperature during the period between flowering and maturity, are the most important factors that influence grain mold development. Warm and humid weather is more conducive to the development of the disease. Humid weather during and after flowering is required for grain mold development and longer the duration of such conditions, more is the incidence of mold development. The severity of fungal sporulation and grain mold increases on most sorghum lines with an increase in the incubation temperature from 25 to 28°C with RH levels of 95–98% (Tonapi et al., 2007). RH, rainfall, the number of rainy days, and minimum temperatures for 4–6 weeks after flowering are significantly correlated with mold incidence (Ratnadassa et al., 2003; Tarekegn et al., 2006).

Grain mold resistance is complex as it involves many fungi (Williams and Rao, 1981) and is governed by many traits (Ambekar et al., 2011). Several management strategies, such as fungicide application, chemical and physical treatments, and the adaptation of tolerant varieties, were tried to control grain mold infection (Navi et al., 2002; Indira et al., 2006). Among all methods, the use of resistant varieties is cost-effective and ecofriendly to control grain mold (Mofokeng et al., 2017). It has been reported by many authors that the resistance to grain mold is conferred by several physical and chemical properties of plant and grains, which include loose panicle architecture, red pericarp, hard grain with increased glume coverage, glume color, pigmented testa, phenolic compounds, high levels of condensed tannin, phenolic acids, flavan-4-ols, and antifungal proteins (Glueck and Rooney, 1980; Esele et al., 1993; Rodriguez-Herrera et al., 1999; Waniska et al., 2001; Audilakshmi et al., 2005; Ulaganathan, 2011; Little et al., 2012; Thirumala Rao et al., 2012). The effects of different plant characters, such as phenological traits (height and duration), panicle structure (compactness), and floral traits [glume cover (GC) and length], on grain mold resistance have been extensively explored. Grain mold resistance is under the control of polygenes involving both major and minor genes having additive and epistatic effects and significant genotype × enviornment interactions (GEI; Klein et al., 2001; Audilakshmi et al., 2005). The involvement of a minimum of 4–10 genes was reported by Rodriguez-Herrera et al. (2000). Due to the complex nature of grain mold, its management has also been difficult.

A grain mold-resistant cultivar provides the best means for minimizing the yield losses due to the disease epidemic (Forbes et al., 1992; Prom and Erpelding, 2009; Prom et al., 2014). This approach is highly recommended in exploring host-plant resistance mechanism. However, most of the efforts in developing mold-resistant cultivars met with challenges to tackle with the complex nature of the fungal species involved, many mechanisms governing resistance, multiple genes involved in genetic resistance, and a significant impact of environment and thus could derive limited success in the development of mold-resistant genotypes (Prom et al., 2003; Audilakshmi et al., 2011; Mpofu and McLaren, 2014). Nevertheless, many efforts are being made to improve grain mold resistance in sorghum. Both conventional breeding approaches and molecular approaches have been followed to tackle the problem of grain mold. Mainly, pedigree breeding was followed by involving resistant sources in the crossing program and making selections in segregating populations. However, major breeding efforts in the last three to four decades to incorporate grain mold resistance in the high-yielding genetic background have not paid many dividends, the real challenge being the incorporation of different resistant mechanisms into a single cultivar to make it mold resistant. Low-to-moderate effect quantitative trait loci (QTLs) conferring the resistance to grain mold were identified in QTL mapping studies (Klein et al., 2001). However, these QTLs did not have a major effect on grain mold resistance directly and were associated with the traits that indirectly modulate resistance. Recently, genome-wide association mapping (GWAS) studies identified a major grain mold-resistant locus containing tightly linked and sequence-related MYB transcription factor genes (Nida et al., 2019; Prom et al., 2020).

A population breeding approach was initiated in Indian Institute of Millets Research (IIMR) as one of the approaches to tackle the problem of grain mold. Population improvement methods provide an opportunity for obtaining desirable gene recombinants by breaking undesirable linkages between desirable and undesirable traits especially for the incorporation of biotic stress- and abiotic stress-resistant genes from agronomically non-elite germplasm lines into the elite background (Reddy et al., 2006). Population improvement involves two important steps. In the first step, broad genetic-based gene pools are created. Later on, they are further improved through recurrent selection methods, which involve a cyclic scheme that includes the selection of desired plants, and a selected recombination, which allows random mating among the selected plants, and the generation of different types of families/progenies from the selected plants. The superior ones are intercrossed for an increased recombination to constitute new populations. The repeated selection and crossing of desirable plants through the recurrent selection methods result in the release of concealed genetic variation due to increased opportunities for recombination, because of which the frequencies of favorable genotypes steadily increase in a population. Population improvement programs have been made feasible in sorghum through the discovery of genetic male sterility. The reciprocal recurrent selection methods are further used to exploit additive (A) and A × A and other epistatic gene actions (Comstock and Robinson, 1952; Eberhart, 1972). Many breeders have adopted the population improvement methods in sorghum (Doggett, 1972; Maunder, 1972). Of several sources of genetic male sterility, the ms3 and ms7 alleles have been extensively used in population improvement programs worldwide due to their stability across environments (Bhola, 1982, Reddy and Stenhouse, 1994; Murty and Rao, 1997). Of late, Bernardino et al. (2021) reported that a multiparental random mating population in sorghum could be used to detect QTLs related to tropical soil adaptation, fine mapping of underlying genes, and genomic selection approaches. Keeping the advantages of the population breeding in bringing together the desirable alleles, we initiated a population breeding approach in 2000 and the two populations were developed, one for female parent (B line) development and another for male parent (R) development. After completing three cycles of random mating and three cycles of half-sib family selections, the lines were stabilized. In the present study, 150 derivatives from a random mating population were screened for their grain mold resistance across the four locations. From these, 33 derivatives were selected and were evaluated for their yield traits and grain mold resistance over the four locations.

The GEI poses a challenge to the plant breeders during testing and the selection of desirable genotypes, resulting in slowing down of the genetic progress (Romagosa and Fox, 1993). A significant GEI may be the resultant of (i) a noncrossover GEI wherein the genotype rank does not significantly change across environments and a significant interaction is mainly due to the magnitude of the response of genotypes with a location, or (ii) a crossover type of GEI, where the rank of the tested genotype changes from one environment to another, which means that a genotype that is best performing in one environment may be a poor performer in another environment. Selection becomes easy for a plant breeder if the GEI is of non-crossover type while the crossover interactions pose difficulties in plant breeding (Mohammadi and Amri, 2013). In such situations, multi-environment trials (METs) help to identify the superior genotypes with broad as well as specific adaptation. The performance of genotypes across environments can be used to derive the relation among the testing locations and also group the locations into different mega-environments (MEs; Yan et al., 2000). Each ME consists of a group of testing locations that show a similar or non-crossover genotypic response as well as depict consistent performance of genotypes across years (Gauch and Zobel, 1997; Yan and Rajcan, 2002; Yan and Tinker, 2005). For interpreting the MET data, the GGE biplot developed by Yan et al. (2000) is preferred by researchers across the world as it allows a visual examination of the GEI pattern. The advantage of this method is that the genotype variance is integrated with the GEI effects removing the only variance due to E. The “which-won-where” view of the GGE biplot identifies the best performing genotype in a particular ME (Yan et al., 2007). In addition, for the most ideal genotype, best suitable environments can be visualized apart from a comparison of any two cultivars across environments, the determination of the yield, and the stability of genotypes in “mean vs. stability view.” An ideal environment for initial testing can be identified by looking into discriminability and representativeness of the environments apart from grouping the locations into MEs (Malla et al., 2010).

The association among the traits across locations can be viewed from the genotype-trait (GT) biplot and an application of the GGE biplot technique where traits are considered as testers (Yan and Rajcan, 2002; Yan and Tinker, 2005; Dehghani et al., 2008). The GT biplots summarize the genotype-by-trait matrix graphically (Yan and Rajcan, 2002; Yan and Kang, 2003). And the graph can also identify the specific trait combinations to be employed for indirect selection (Yan and Tinker, 2005). In addition, the potential known genotypes that are good performers for specific traits give insights into a parental line with trait combinations for enhancing yield. The GGE biplot technique was used to understand the stability of genotypes and also to interpret the complexity of GEI in grain sorghum (Rakshit et al., 2012), forage sorghum (Aruna et al., 2016), and sweet sorghum (Rao et al., 2011).

The present study aims to evaluate the performance and stability of 33 derivatives from the population improvement program for grain mold resistance and GY across the four locations. The main objectives were (i) to interpret the complexity of GEI in GM using the GGE biplot analysis; (ii) to identify stable lines with the grain mold resistance that could be utilized in the resistance breeding programs; and (iii) to understand trait associations among grain mold traits using the GT biplot analysis.

## Materials and Methods

### Plant Material

The plant material consists of the derivatives of the population breeding approach. In the early 2000s, population breeding has been initiated at ICAR-IIMR to facilitate the accumulation of genes from different resistance sources to tackle the grain mold problem using a half-sib mating system. Two separate populations were maintained, one for B line and another for R line improvement. Care was taken to involve a different set of lines in each of the populations to maintain genetic diversity. In the B population, elite B lines (296B, 27B, 422B, and 463B), some elite sources of grain mold resistance (SPGM 950267, SPGM 950283, and SPGM 950288), and grain mold-resistant sources from germplasm (B 58586 and IS 25017) were used. In the R population, elite R lines (C43, C85, NR 9, and NR 486), improved the grain mold-resistant source (SR 839) and the resistant source from germplasm (IS 14332), were involved. Three cycles of random mating were allowed in each of the populations in isolation. Later, three cycles of selections through half-sib mating were carried out. Each cycle of half-sib mating consists of one generation of selection for the target trait and one generation of random mating among the selected lines, thus increasing the frequency of desirable alleles. After three cycles of half-sib mating, the population mean of grain mold had come down to 5.6 score (on the scale of 1–9) from an initial score of 8.0 in case of the B line population, whereas it had come down to 5.4 from an initial score of 7.0 in case of the R line population. After three cycles of half-sib mating, the lines were made genetically stable by selfing over 3–4 generations. A total of 150 such stable lines were evaluated in the four locations during 2017. Out of these, 33 derivatives that performed well across the locations were selected and again tested in the locations during 2018.

### Experimental Location and Design

**Figure 1:**
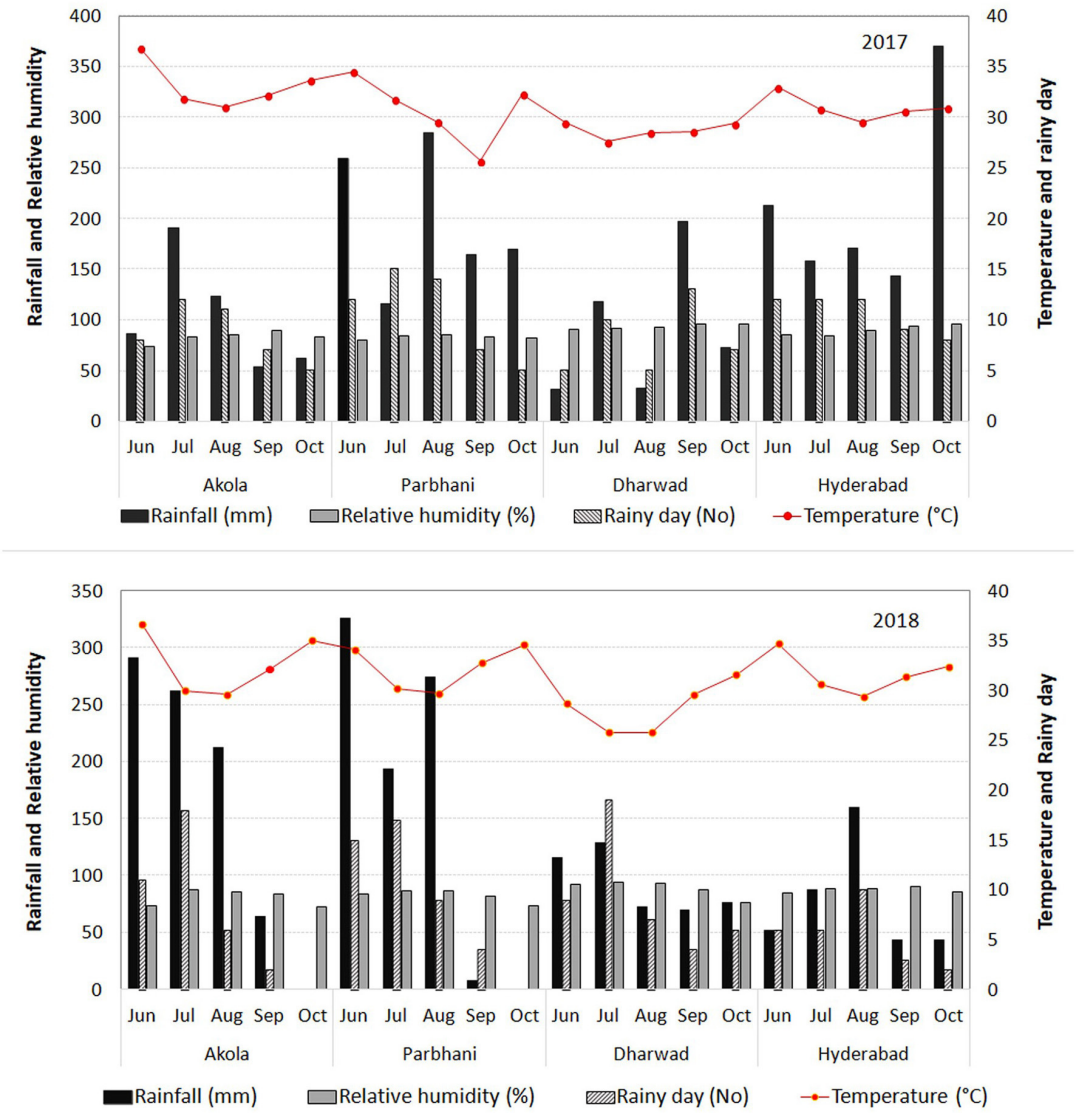
Meteorological data [rainfall, the number of rainy days, relative humidity (RH), and temperature] in the four locations of the study during 2017 and 2018.

The trials were conducted in the four testing locations during 2017 and 2018. The chosen locations were from sorghum growing states such as Maharashtra, Karnataka, and Telangana. These locations were distributed across the three states of India, with two locations (Akola and Parbhani) in Maharashtra, one each in Karnataka (Dharwad) and Telangana (Hyderabad). All these four locations were earlier identified as hot-spot locations for sorghum grain mold under All India Co-ordinated Research Project on Sorghum (AICRP-Sorghum). The climatic conditions of the selected locations for this study are presented in Figure 1.

In 2017, 150 random mating-derived lines were sown in an augmented design in the four locations. These lines were shown in three blocks each with 50 lines. In each block, five checks were included. The checks include two high-yielding but grain mold-susceptible B lines (296B and 27B), two moderately grain mold-resistant R lines (C43 and CB33), and one grain mold-resistant check (B 58586). In 2018, the experimental design was a randomized complete block (RCBD) with three replications. Two checks, one grain mold-resistant check, B 58586 and an elite grain mold-susceptible check, 296B were used for the experiment. Each genotype had a plot size of two rows of 4 m length each, with 0.6 m between rows and 0.15 m between hills in each row. In each location, the experiment was planted with the onset of rains during June. All the recommended crop production and protection practices were followed to raise a crop with good plant stand. Standard crop management practices were followed across all locations.

### Field Monitoring and Data Collection

In 2017, observations on grain mold score, days to flower (DF), plant height (PH), GY per plant, and 100-grain weight (HGW) were recorded, whereas, in 2018, all the following observations were recorded in each location.

#### Phenological and Yield Traits

##### Days to Flower

Number of days to anthesis for ~50% of the plants to reach mid bloom was recorded for each plot.

##### Plant Height

PH was recorded at physiological maturity on 10 plants per plot, from the soil surface to the top of the panicle and was expressed in cm.

##### Panicle Length

The length of the panicle from the base to the tip was measured on 10 plants per plot at the time of harvest and was expressed in cm.

##### Grain Yield

The grain of 10 plants was harvested individually and weighted. The average weight of the 10 plants was taken as the yield per plant in grams. The first and last plants in each row were not harvested to eliminate the confounding results caused by a border effect.

##### Hundred-Grain Weight

The weight of 100 grains from each panicle was recorded and expressed in grams. The average of 10 plants is used for analysis.

##### Grain Hardness

Digital hardness tester (Pharmag Instruments Ltd., Kutkatpally, Hyderabad, India) was used to record grain hardness (GHR) for the grain samples from Hyderabad and Akola. It employed a force gauge to measure tension/compression force applied on an individual seed. The measurement on 10 randomly selected seeds per plant and on 5 plants per replication was taken. The individual seed hardness from each sample was tested, and the mean value was expressed in kilogram force (kgf).

##### Grain Mold Score

Threshing of grains were carried out plot-wise, and the grain mold reactions of each plot were measured as grain mold score (GMS) as per Audilakshmi et al. (2011). About 20 g of the grain sample was placed in a petri plate, and grain mold infection was scored visually using a 1–9 scale, where 1 = no mold and 9 = more than 75% of grains in the sample are with mold. The GMS data were subjected to square root transformation for further analysis.

##### Glume Cover

GC was assessed visually based on the portion of the grain covered with glume as 25, 50, 75, and 100%. This visual assessment was taken on 10 plants in each plot and the average was drawn. The data were transformed using an arcsine transformation.

##### Glume Color

GCL was recorded visually as white, red, and brown on 10 plants in each plot.

##### Panicle Compactness

Panicle compactness (PC) is recorded visually as loose, semi-compact, and compact based on the density of the ear head at the time of maturity.

### Data Analysis

The 2017 data of all the four locations were subjected to the individual and pooled augmented analysis through the augmented complete block design (ACBD) in R program (Rodríguez et al., 2018). Out of 150 genotypes tested, 33 superior derivatives were selected for evaluation in 2018 based on their grain mold score across the locations in 2017. For the data of the four locations in 2018, ANOVA was carried out to test location (L), genotype (G), and their interaction effects (G × L). Trait variability and correlations were calculated using replicate means. The replication-wise data were analyzed for ANOVA using Genstat 12th edition (V. S. N., International, 2009). Heritability (h^2^b) is calculated for the target traits as the ratio of genetic variance to the phenotypic variance (which is the total of the genetic variance and environmental variance). The levels of the broad-sense heritability (h^2^bs) are categorized as low (<0.3), moderate (0.3–0.6), and high (>0.6) according to Robinson et al. (1949).

The GGE biplot analysis was employed to interpret the genotype by environment interactions for GY and grain mold tolerance. A GGE biplot methodology, which explains two concepts, the biplot concept (Gabriel, 1971) and the GGE concept (Yan et al., 2000), was used to analyze the data. The statistical theory of GGE methodology as given by Yan and Kang (2003) was considered. The data were analyzed as described by Rakshit et al. (2012) using the software GGE biplot ver. 8.2 (Yan, 2001). The MLT data were analyzed in the GGE biplot with the options (scaling = “scaling 4”) and tester-centered (centering = 2) GGE biplot, as suggested by Yan and Holland (2010). For obtaining genotypes with high yield and stability using the “mean vs. stability” option, genotype-focused singular value partitioning (SVP = 1) was used, and for evaluating locations, environment-focused SVP = 2 was employed (Yan, 2001) using the “relation among testers” option. The “which-won-where” option was used to pool the locations into MEs and to identify the promising genotype in a given ME.

#### Genotype-By-Trait Biplot

The GGE biplot software was used to generate genotype × trait biplots by the “genotype-by-trait biplots” option using the pooled data and “scaling = 1.” Traits were considered as testers. Phenotypic correlations among traits were determined using trait-focused SVP (Yan and Tinker, 2005).

## Results

### ANOVA and Mean Performance

The mean values and ranges of trait expression of the studied 150 genotypes in 2017 are presented as location wise in Table 1. The grain mold incidence was high in Hyderabad and Dharwad as revealed by the location mean and range of grain mold score. Good amount of variation was observed for all other traits. A total of 33 derivatives were selected based on their better level of grain mold resistance across the four locations for further study during 2018. The results of ANOVA and the mean values for all the traits studied during 2018 are presented in Tables 2, 3. The ANOVA showed that the effects of G and L and G × L interaction effects were significant (*p* < 0.01) for all traits. The DF ranged from 66 to 80 days with G6, G7, G8, G5, G15, and G13 flowering in <70 days. It was longer in Akola, whereas it was shorter in Hyderabad. There was a significant effect of G, L, and the interaction G × L on flowering. The genotypes varied significantly for PH, which ranged from 123 to 223 cm. PH observed at Dharwad and Hyderabad was higher compared to that observed at Akola and Parbhani. For GY also, the differences among the genotypes were significant and there are a few genotypes, such as G28, G21, G10, G23, G27, and G1, that showed significantly higher performance for GY per plant over the elite check, 296B. In general, GY was less at Akola and Parbhani. The lines, G15, G28, and G7 exhibited significantly higher HGW over the elite check, 296B. The differences in grain weight among the locations was not much. Most genotypes recorded higher grain mold score in 2017 compared to 2018. For this trait, the genotypes G4, G33, G31, and G8 were statistically on par with the resistant check, B 58586 in both the years. However, all other test lines were significantly better than the elite check, 296B. Their GMS ranged from 2.78 to 5.54 in 2018, which indicates that the lines were all tolerant. The elite parent 296B had a GMS of 8.5 in 2017 and 7.75 in 2018 indicating that it is susceptible. The highest GMS among the locations was recorded at Hyderabad (6.8) followed by Dharwad (5.87) in both years. Though there were significant differences among locations, all the genotypes were categorized as tolerant based on the observed GMS values at these locations. GHR, which was measured at two locations, ranged from 6.33 to 12.33 kgf. The grain samples of Akola were harder compared to those of Hyderabad.

**Table 1:** Mean performance of all genotypes at different locations during the rainy season of 2017.

**Centre**	**Trait**	**Range**	**Mean**	**Lsd (5%)**
Akola	DF	57–92	70.1	7.82
	GMS	1.8–7.74	4.0	1.71
	PH	93.9–343.5	163.8	15.59
	HGW	1.04–3.31	2.35	0.37
	GY	8.87–45.9	22.26	12.13
Parbhani	DF	53–73	59	5.05
	GMS	1.93–8.27	4.88	1.66
	PH	96–403	191.9	0.004
	HGW	1.01–5.20	2.01	0.68
	GY	6.73–88.5	52.9	22.17
Dharwad	DF	57–74	67.0	2.86
	GMS	2.74–8.24	5.87	2.12
	PH	81.7–323.3	171.8	49.6
	HGW	1.50–3.59	2.24	0.50
	GY	9.94–62.9	35.34	14.36
Hyderabad	DF	54–81	70.2	6.66
	GMS	2.30–9.16	6.8	1.67
	PH	98.5–326.1	167.7	29.6
	HGW	1.27–3.73	2.37	0.52
	GY	8.91–87.7	39.59	14.68
Across	DF	63–76	68.8	4.29
	GMS	2.99–7.55	5.56	1.32
	PH	119.4–270.3	173.8	38.83
	HGW	1.79–3.13	2.24	0.38
	GY	25.62–49.74	37.55	11.84

*DF, Days to flower; PH, Plant height (cm); HGW, 100-grain weight (g); GY, Grain yield (g/plant); GMS, Grain mold score*.

**Table 2:** **(A)** ANOVA for different studied traits across four locations during the rainy season of 2018. **(B)** ANOVA for grain yield and grain mold score in individual locations during rainy season of 2018.

**(A)**
	**df**	**DF**	**PH**	**GC**	**PL**	**GY**	**HGW**	**GMS**	**df**	**GH**
Genotype	34	126.2^**^	7538.7^**^	1919.75^**^	74.21^**^	912.9^**^	0.63^**^	6.56^**^	34	2.28^**^
Location	3	2113.67^**^	70544.1^**^	2488.41^**^	206.9^**^	47670^**^	4.62^**^	31.98^**^	1	660.4^**^
Genotype × Location	102	34.26^**^	1588.1^**^	575.34^**^	25.43^*^	594.7^**^	0.36^**^	2.89^**^	34	2.20^**^
Residual	278	14.87	101.1	85.93	18.17	150	0.09	1.241	138	0.04
**(B)**
**Source**		**Grain yield**				**Grain mold score**			
	**df**	**Akola**	**Dharwad**	**Hyderabad**	**Parbhani**	**Akola**	**Dharwad**	**Hyderabad**	**Parbhani**
Replication	2	79.4	558.9	14.65	41.3	35.3	8.15	0.43	0.44
Genotype	34	123.2^**^	1907.2^**^	500.2^**^	166.3^*^	2.01^**^	7.17^**^	3.78^**^	1.54^*^
Residual	68	25.1	384.9	46.0	145.4	0.42	3.00	0.64	1.24

* *p < 0.05;*

** *p < 0.01; DF, Days to flower; PH, Plant height; GC, Glume cover; PL, Panicle length; GY, Grain yield; HGW, 100-grain weight; GMS, Grain mold score; GHR, Grain hardness*.

**Table 3:** *Per se* performance of genotypes for all traits across locations during the rainy seasons of 2017 and 2018.

**Genotype**	**DF**	**PH (cm)**	**GC (%)**	**PL (cm)**	**GY (g/pl)**	**HGW (g)**	**GMS**		**GH (kgf)**
							**2017**	**2018**	
G1	78	189	67	31.5	53.06	2.50	4.18	4.43	12.33
G2	80	123	49	23.1	34.58	2.06	4.74	4.58	7.83
G3	78	144	68	31.3	44.48	1.90	5.09	3.49	7.67
G4	72	198	61	27.6	35.29	2.30	3.95	2.78	7.83
G5	68	172	41	23.1	39.71	2.34	5.18	3.80	8.5
G6	66	163	61	25.9	34.92	2.42	5.41	3.93	10.17
G7	67	139	55	24.0	32.93	2.44	5.70	5.37	6.83
G8	67	166	50	29.3	52.35	2.59	3.67	3.40	9.83
G9	77	170	49	27.6	42.50	2.38	4.03	3.43	9.17
G10	72	207	49	25.5	58.08	2.13	5.14	4.35	6.33
G11	77	174	48	27.9	45.94	2.28	4.54	4.82	7.17
G12	73	223	44	29.5	46.96	2.24	4.05	3.47	9.67
G13	70	148	52	24.4	37.19	2.24	5.97	4.32	7.33
G14	72	192	68	31.2	43.67	2.17	5.18	4.66	8.33
G15	69	183	52	26.7	37.00	2.71	5.61	4.57	10.33
G16	74	141	54	26.9	47.31	2.35	5.21	4.19	9.17
G17	76	132	35	27.7	43.73	2.47	5.34	5.03	8.67
G18	74	155	41	27.0	40.49	2.31	5.61	4.35	7.83
G19	80	154	30	27.5	45.17	2.43	4.68	4.78	8.67
G20	71	179	31	23.2	37.08	2.34	4.61	4.24	9.17
G21	76	152	70	23.7	61.29	2.36	4.71	4.57	9.33
G22	79	182	48	28.2	46.94	2.15	5.24	4.33	8.0
G23	77	174	57	25.1	55.63	2.36	4.12	3.83	7.33
G24	77	169	28	22.2	47.77	1.71	5.21	4.34	8.33
G25	77	180	45	27.0	49.88	1.89	4.14	3.61	9.17
G26	73	221	50	26.8	47.77	2.41	3.91	3.74	7.33
G27	79	198	56	23.5	53.83	2.36	3.85	3.56	9.17
G28	80	191	51	23.1	71.58	2.69	4.74	4.17	7.17
G29	75	145	42	26.3	33.98	2.21	3.75	3.87	8.33
G30	79	159	45	25.7	49.94	2.38	5.41	5.54	9.17
G31	75	150	51	26.7	48.52	2.39	4.06	3.31	8.33
G32	74	158	57	22.8	48.55	2.53	4.43	4.11	10.17
G33	76	156	65	26.8	31.90	2.02	3.50	2.93	9.83
G34 (B 58586)	79	202	90	27.6	38.88	1.78	2.89	2.52	9.67
G35 (296B)	79	134	36	27.8	42.75	2.35	8.50	7.75	7.83
**Location**
Akola	80.4	154	56	25.0	25.29	2.17	4.0	3.43	8.63
Dharward	75.4	192	51	25.5	66.82	2.43	5.87	4.20	-
Hyderabad	69.4	190	45	28.1	60.08	2.50	6.8	4.77	6.08
Parbhani	75.5	141	53	27.0	28.57	2.25	4.88	4.08	-
**Grand mean**	75	169	51	26.4	45.19	2.29	5.39	4.12	6.86
**Lsd (5%)**	3.10	8.08	7.45	3.40	9.84	0.24	1.32	0.90	0.23
**CV**	5.1	5.9	18.1	16.1	27.1	13.0	-	27.0	2.9

### Broad-Sense Heritability

The h^2^bs for the yield and grain mold-resistant traits for an individual location and across the locations is presented in Table 4. Although the observed h^2^b was generally high for most of the traits, it showed a variation for each trait in respective locations. H^2^b was high for days to flowering, PH, GC, and HGW in all locations and across locations. GY had high h^2^b at Akola (0.8), Dharwad (0.8), and Hyderabad (0.91), whereas it had low h^2^b at Parbhani (0.13). Across locations, it was found to have moderate h^2^b (0.35). Grain mold score had moderate h^2^b at Akola (0.53) and Dharwad (0.6), whereas it showed high h^2^b at Hyderabad (0.83). Parbhani recorded low h^2^b for grain mold score (0.19). GHR recorded moderate h^2^b in both the locations where it was recorded.

**Table 4:** Broad-sense heritability (h^2^bs) for the traits location wise and across locations during the rainy season of 2018.

**Trait/Location**	**Akola**	**Dharward**	**Hyderabad**	**Parbhani**	**Pooled data**
Days flower	0.98	0.98	0.88	NE^**^	0.73
Plant height	0.97	0.99	0.97	0.91	0.79
Glume cover	0.80	0.82	0.93	0.84	0.7
Panicle length	0.76	0.44	0.89	0.03	0.66
Grain yield per plant	0.8	0.8	0.91	0.13	0.35
100 grain weight	0.97	0.81	0.86	0.75	0.63
Grain mold score	0.53	0.6	0.83	0.19	0.56
Grain hardness	0.58	NA^*^	0.38	NA^*^	0.40

* *NA indicates trait values are not measured in this year for this location*.

** *NE indicated that heritability (h^2^b) is not calculated because their variance is negative*.

### Contribution of the Factors to the Variation of GY and Grain Mold Score

The relative contribution of variation due to genotype, environment, and G × E to the total variance measured for GY and GMS are presented in Table 5. The location with 51.7% of the total variance was the major contributor toward a variation in GY followed by the G × L interaction (21.9%). The genotype accounted for 11.2% of total variability. Similarly, a variation in GMS was largely explained by G × L (30.7%) followed by the G (23.3%) and L (10%).

**Table 5:** Sum of squares and proportion of variation explained by year (Y), location (L), genotype (G), and their interactions on grain yield (GY) and grain mold score (GMS).

	**df**	**Grain yield**		**GMS**	
		**Sum of squares**	**%ssT**	**Sum of squares**	**%ssT**
Genotype	34	31038.3	11.2	223.1	23.3
Location	3	143009.9	51.7	95.9	10.0
Genotype × Location	102	60655.4	21.9	294.5	30.7
Residual	278	41688.4	15.1	345.0	36.0
Total		276392		958.6	

### GGE Biplot Analysis

For ease of interpretation of a graphical representation, grain mold score was rearranged with scaling 9 = resistant and 1 = susceptible for the GGE biplot analysis. Because the heritability was different in different environments, the GGE biplot analysis was done by applying h^2^b-adjusted GGE as suggested by Yan and Holland (2010).

#### Mean Performance and Stability Analysis of Genotypes Across Locations

**Figure 2:**
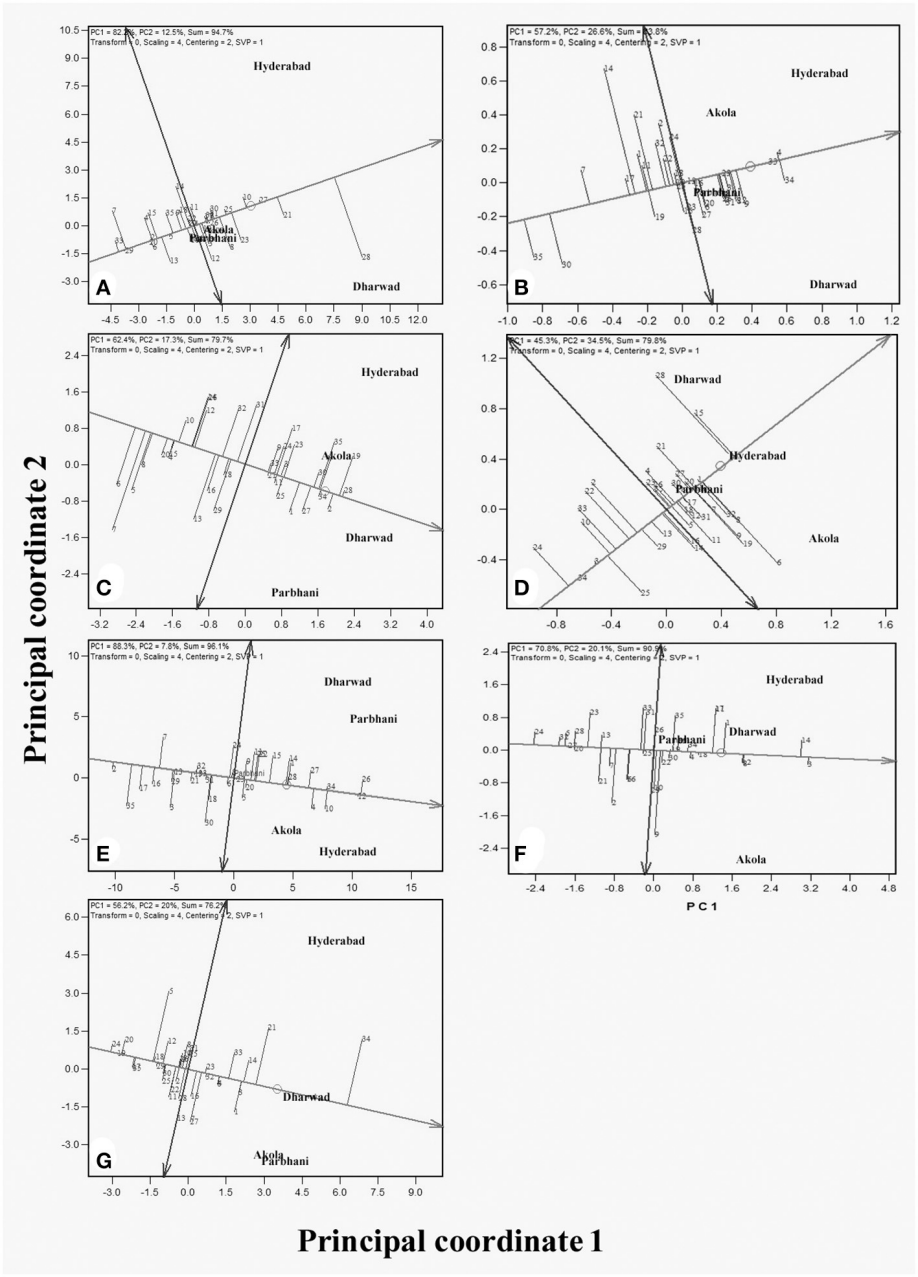
GGE biplots from the combined analysis of data from 35 genotypes (1–35) evaluated across four locations for the traits: **(A)** grain yield (GY), **(B)** grain mold score, **(C)** flowering time, **(D)** 100-grain weight (HGW), **(E)** plant height (PH), **(F)** panicle length (PL), and **(G)** glume coverage.

The performance of genotypes for mean and stability is depicted by the average environment coordination (AEC) method and is presented in Figure 2 (Yan, 2001, 2002). The environment-centered (centering = 2) genotype-metric (SVP = 1) biplots for each of the traits are presented in Figures 2A–G. The first two PCs explained about 94.7% of a variation for GY. In Figure 2 the AEC abscissa, a line with a single arrow head, passes through the biplot origin and points toward the higher mean values. The perpendicular lines to the AEC abscissa are the AEC ordinates and their length indicates stability. The genotype, which is present away from the origin toward the arrow head, has the highest and in the opposite direction has the lowest mean GY. Accordingly, genotype G28 which is placed on the positive side at the far end from the origin, is the highest yielding, followed by G21. On the other hand, G33 had the lowest GY, followed by G7. The biplot results were compared with those of tabulated values for mean GYs of the genotypes (Table 3), thereby showing that the biplot can be used to visualize ranking of genotypes. The projection of the genotypes on the AEC ordinate approximated the stability with a greater projection indicating toward instability. The genotype G28 was less stable for GY with a higher projection from the AEC abscissa. On the contrary, G21 was relatively more stable as it was closer to the AEC abscissa. It also had the highest GY after G28.

For grain mold score, the first two PCs explained 83.8% of the variation. Here, the resistant genotype (G34) had the best level of resistance, being projected farthest from the origin. The genotypes, G4 and G33 had good levels of grain mold resistance and were more stable as their projection on the AEC ordinate is less. Though G8 and G9 also had low grain mold scores, they are not very stable.

Grain yield and grain mold resistance can be simultaneously improved indirectly through other target traits. The mean and stability biplots for all these traits are presented in Figures 2C–G. The analysis depicts desirable genotypes for each of these traits. The first two PCs explained 76.2–96.1% of the total variation in the data for different traits, thereby these graphs can be used for the interpretation of MLT data. For days to flowering, PC1 and PC2 explained 79.7% of the total variation and G6 was the earliest genotype with 66 DF, followed by G7 and G5. For PH, the biplot explained 96.1% of the variation. The genotypes G12 and G26 are the tallest and G2 is the shortest. The biplot of panicle length (PL) explained 90.9% of the total variation and G3 and G14 had longer panicles. For HGW, the biplot explained 79.8% of the variation and the genotypes, G28 and G15 had a bold seed with the grain weight of around 2.7 g/100 grain. The biplot of glume coverage explained 76.2% of the total variation. The longest glumes were observed in the resistant genotype (G34). Among the test lines, G21, G3, and G14 had longer glumes (68–70%). The numerical values of these traits (Table 3) were correlated with the graphical results, except a few minor changes in genotype ranking.

**Figure 3:**
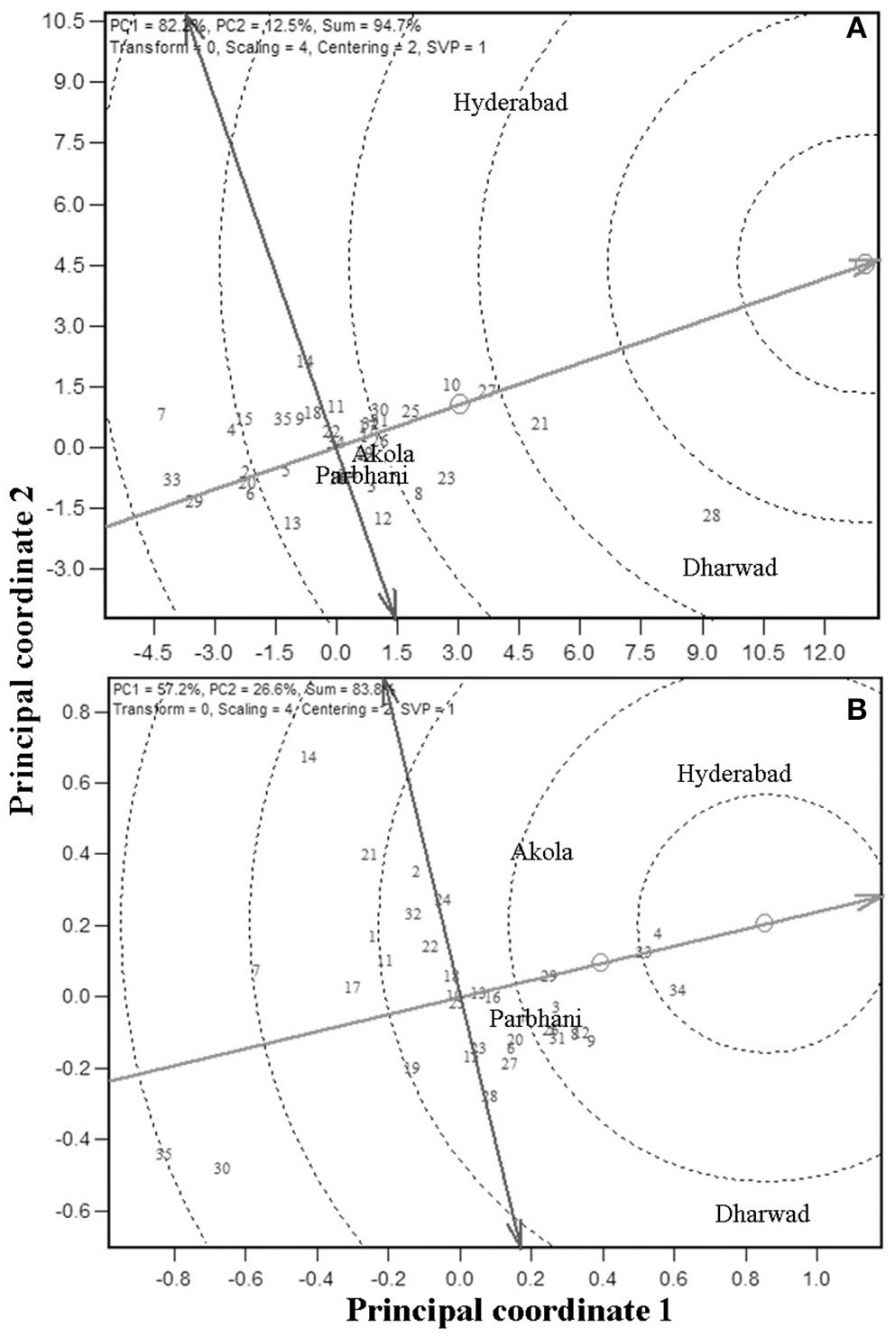
Ranking of 35 genotypes relative to ideal genotype [the small circle on average environment coordinate, average environment coordination (AEC)] for GY **(A)** and GMS **(B)**.

**Figure 4:**
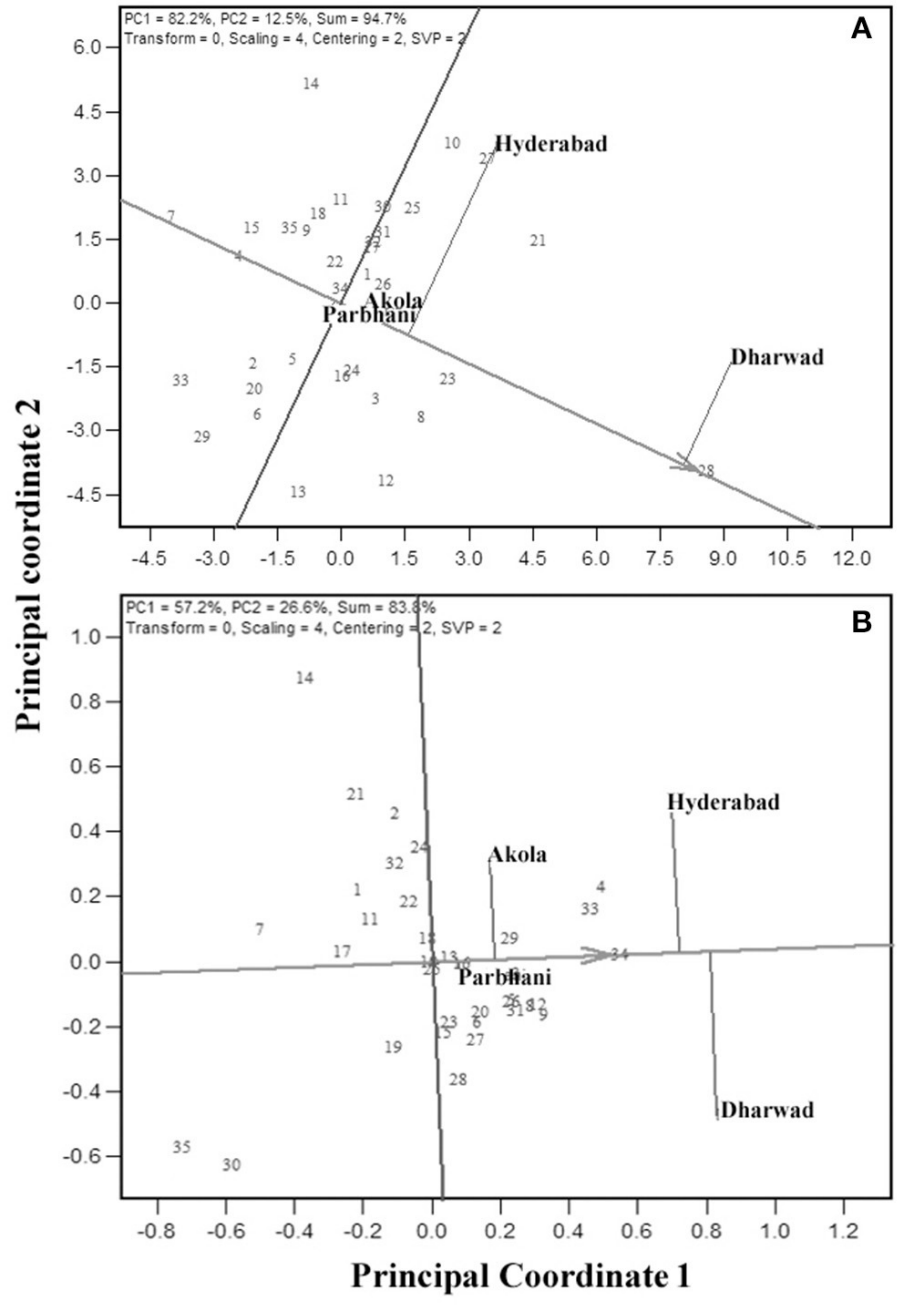
Ranking of four environments based on the performance of the highest yielding genotype, 28 for GY **(A)** and most resistant genotype, 34 for grain mold score **(B)**.

Because GMS and GY are the economically important traits, these traits are considered for further analysis. An “ideal genotype” is defined as one, which has high mean yield and stability across locations. Ranking of the other genotypes in comparison to the “ideal genotype” for GY is presented in Figure 3 (Yan and Tinker, 2006). In the present study, genotypes G28, G21, G27, and G10 were closer to the ideal genotype (Figure 3A) and also had high numerical GY among the tested genotypes (Table 3). For grain mold resistance, G34, G4, and G33 were closer to an ideal one indicating they had a better level of grain mold tolerance. G28 was the highest yielder and was selected to understand the specific adaptation by ranking the test environment based on the relative GY of this genotype in the given environment (Figure 4A). Genotype 28 had the highest yields at Dharwad, above average performance at Hyderabad, near average at Akola, and below average at Parbhani. For grain mold resistance, G34 had a high level of resistance at Dharwad, followed by Hyderabad (Figure 4B). Its performance in all other locations was good and above average.

#### Ideal Test Locations for GY and Grain Mold Resistance

**Figure 5:**
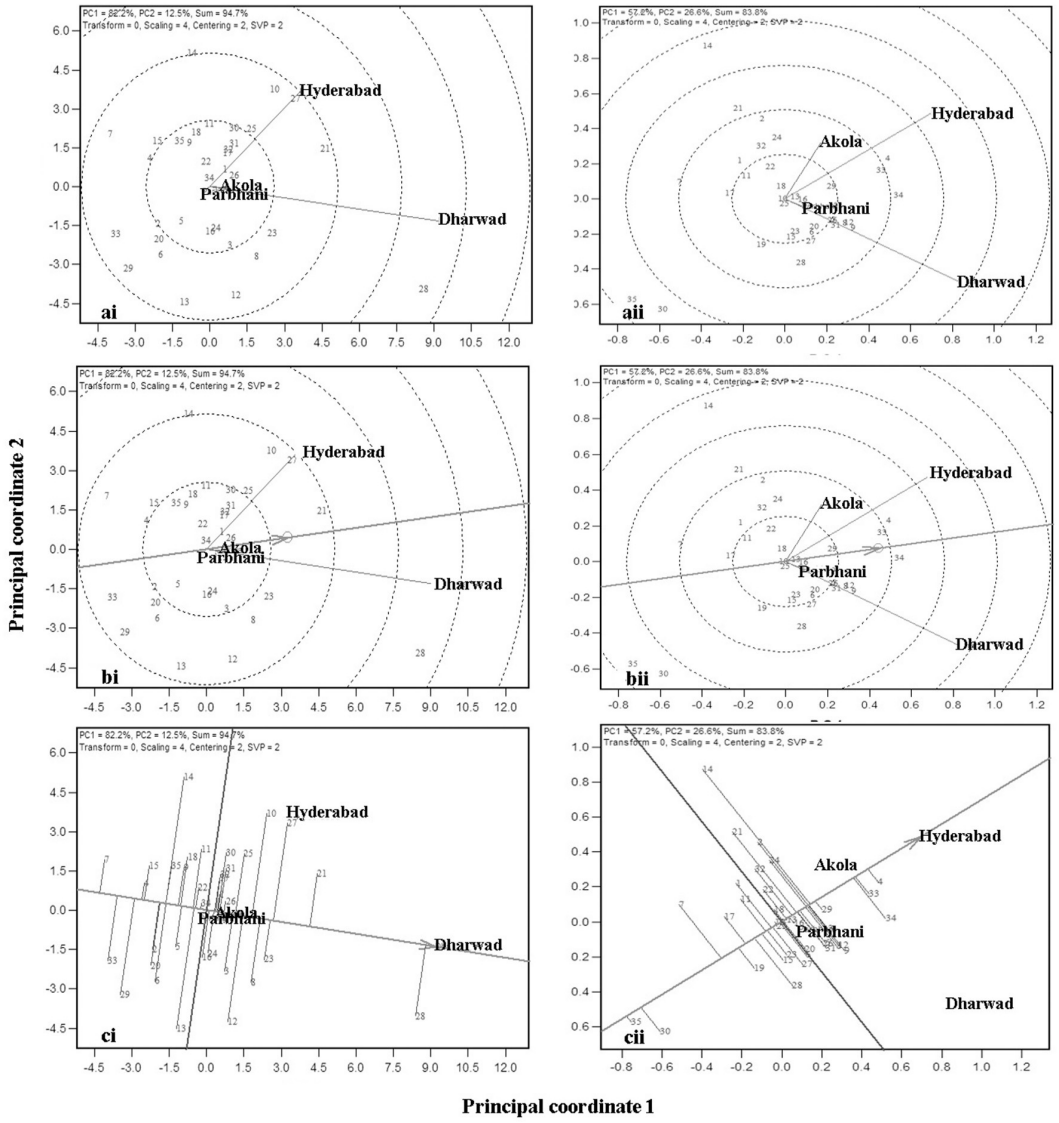
GGE biplot showing relation among the four test locations with respect to GY (**a**i) and grain mold score (**a**ii); ranking of locations based on the discriminating ability and representativeness for GY (**b**i) and grain mold score (**b**ii); and Ranking of 35 sorghum genotypes based on their performance in the near ideal location, for GY (**c**i) and grain mold score (**c**ii).

The MLT data can also be used to derive the information about testing locations (Cooper et al., 1996; Yan et al., 2007). Graphical representation of the test environment can be viewed from the GGE biplot. An ideal environment is one, which can differentiate among the genotypes that can be measured by its vector length in the biplot, and it should represent all other locations which is, measured by its angle with the “average environment” (Yan, 2001). In the GGE biplot, the relationships among test environments can be derived by environment-metric (SVP = 2). The cosine of the angle between the two environments is related to the similarity between them. An acute angle indicates a closer relationship between the environments in ranking the genotypes, whereas an obtuse angle is indicative of a negative correlation and a right angle of no relation (Yan and Tinker, 2006). Combined analysis of the 35 genotypes for GY (Figure 5ai) showed that the locations Hyderabad, Dharwad, and Akola were positively correlated, whereas Parbhani with an obtuse angle with other locations had a negative association. For GMS, Dharwad, Parbhani, and Hyderabad were positively correlated with an acute angle, whereas Akola had no linear correlation with Dharwad and Parbhani but positively correlated with Hyderabad (Figure 5aii). The results of correlations among environments using the GGE biplot for GY and grain mold score were further confirmed by carrying out rank correlations among the environments (Table 6).

**Table 6:** Rank correlation between environments for GY and grain mold score.

**Environment**	**Akola**	**Dharwad**	**Hyderabad**	**Parbhani**
**Grain yield**
Akola	1			
Dharwad	0.295	1		
Hyderabad	0.180	0.572**	1	
Parbhani	−0.155	0.247	−0.382*	1
**Grain mold score**
Akola	1			
Dharwad	0.123	1		
Hyderabad	0.211	0.50**	1	
Parbhani	0.038	0.432*	0.107	1

The discriminating ability and representativeness of the environments are visualized in Figure 5bi,ii for GY and grain mold resistance, respectively. The concentric circles on the biplot are proportional to the SD of the environments. It indicates the discriminating ability of the environments (Yan and Tinker, 2006). In addition, the length of the environment vector is directly related to the discriminating ability. Dharwad with the longest vector length was found to be the most discriminating location, followed by Hyderabad for both GY and grain mold resistance. Parbhani and Akola revealed no discriminating power as depicted by a shorter vector length, and the conclusions on the genotypic performance may not be meaningful. Representativeness of a location can be obtained by projecting the locations with respect to the AEC abscissa (Figure 5b). The “average environment” is represented by a small circle on the AEA in Figure 5b. Those environments, which make smaller angles with the AEA, are the most representative of all the test environments. Thus, Dharwad was closest to the average environment in case of GY, whereas both Hyderabad and Dharwad had almost the same angle in case of grain mold score. An “ideal” location is the one, which should be discriminating as well as represent the test locations (Yan, 2001). Thus, Dharwad emerged as an ideal location for the selection of genotypes with high yield and adaptability. For ranking the genotypes for GY in the near average environment, Dharwad (Figure 5ci) showed that at Dharwad, G28 yielded maximum followed by G21, G27, and G23, whereas G7 followed by G33 recorded the lowest yields. The genotypes, G16 and G22 showed near average GYs. For grain mold resistance at Hyderabad, G4, G33, and G34 showed high levels of resistance, whereas G35 and G30 recorded lower levels of resistance (Figure 5ci).

#### “Which-Won-Where” Analysis and Clustering of Environments

**Figure 6:**
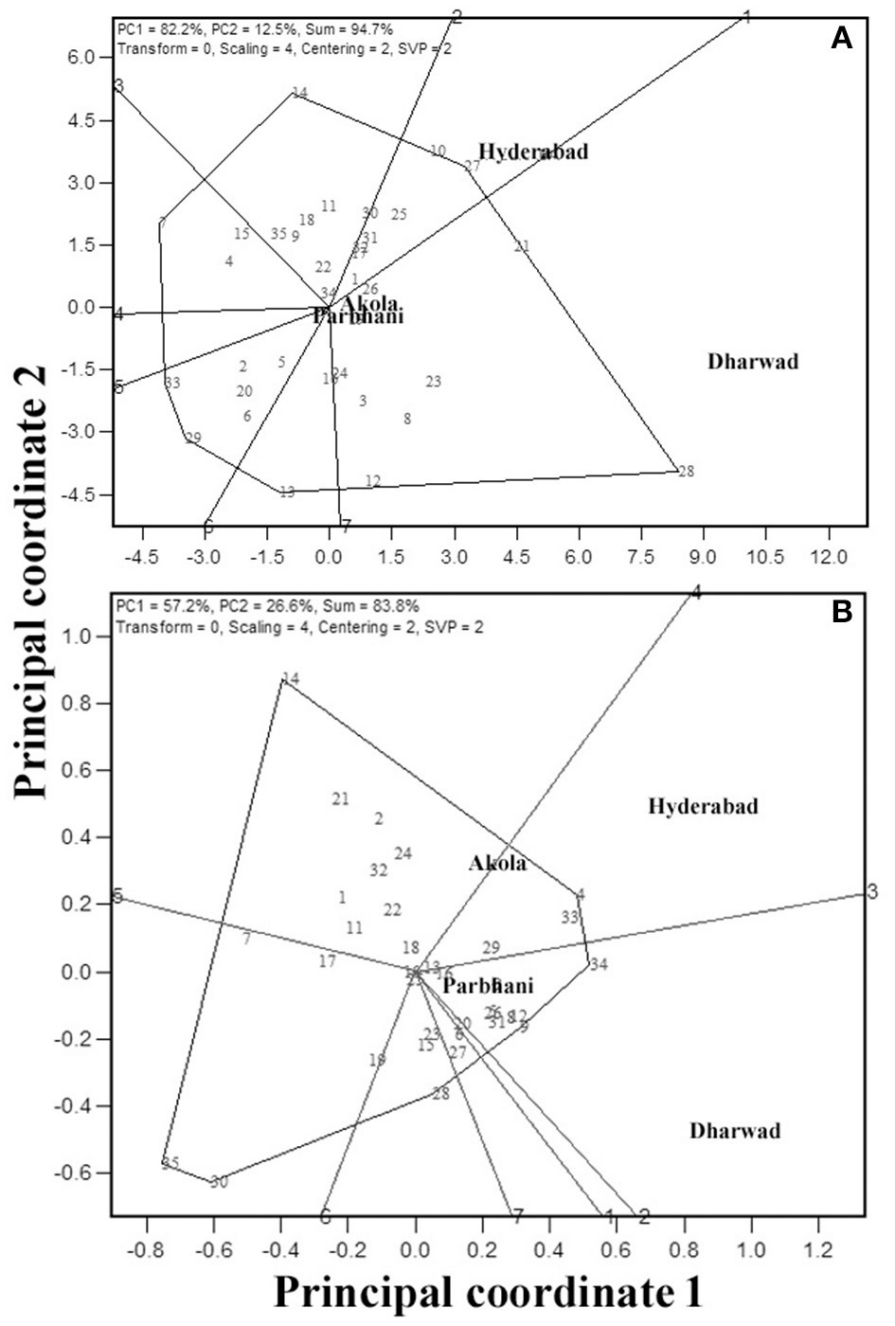
GGE biplot indicating “which-won-where” analysis of the 35 sorghum genotypes for GY **(A)** and grain mold score **(B)**.

“Which-won-where” feature of the GGE biplot is exploited by researchers in many crops to derive the best performing genotypes in a subset of environments. In this view, the genotypes that are far away from the biplot origin are joined to form a polygon, and perpendicular lines are drawn from the origin to each side of the polygon. This splits the polygon into several sectors with a genotype at the vertex. The “Which-won-where” biplot for GY is presented in Figure 6A. The biplot indicated the existence of crossover GEIs and clusters of environments. The polygon had seven genotypes, viz., G28, G27, G14, G7, G33, G29, and G13 at the vertices. The perpendicular lines divided the biplot into seven sectors, of which three of them included all the four locations. Thus, the test locations were divided into three clusters, one with Dharwad and Akola with G28 as the best performing genotype for GY. The second cluster encompassed Hyderabad with G27 as the best genotype. The third cluster had Parbhani with G13 as the best genotype. For GMS, six genotypes, viz., G34, G28, G30, G35, G14, and G4 are placed at the vertices (Figure 6B) of the hexagon. Of the six sectors, two of them included four locations. Thus, the test locations were divided into two clusters. One with Dharwad and Parbhani with G34 as the best genotype. The second cluster had Hyderabad and Akola with G4 as the best genotype.

### Trait Associations Through Genotype-By-Trait Biplot

**Figure 7:**
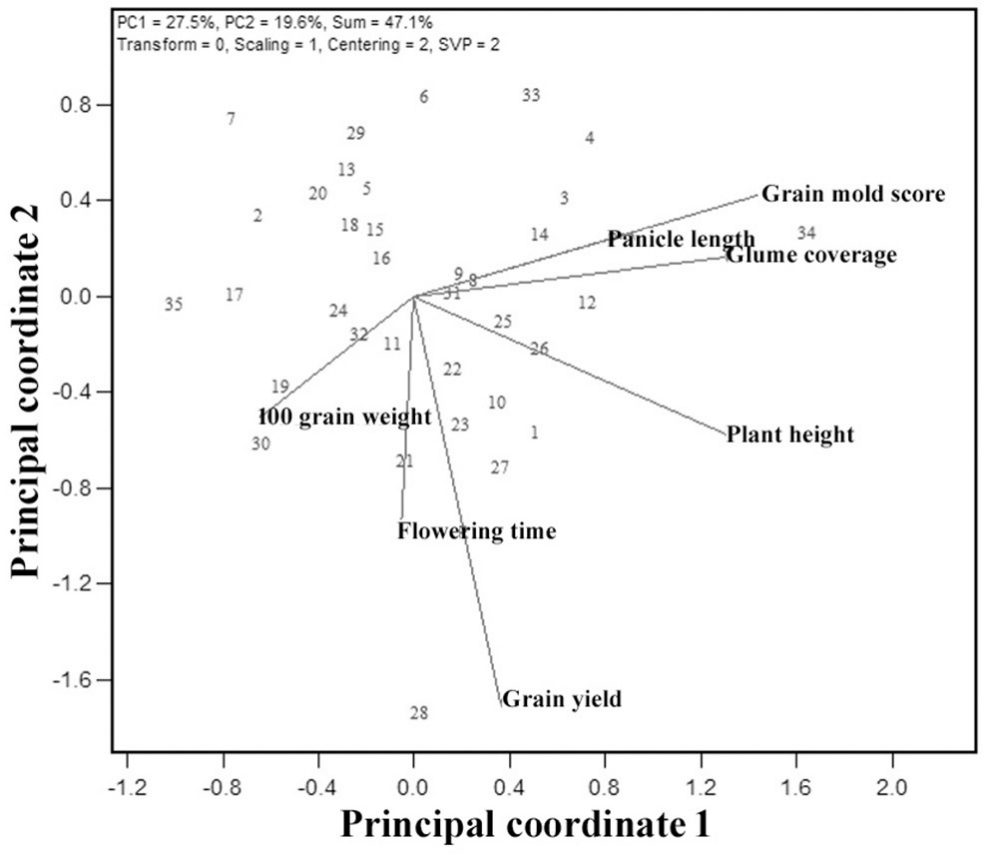
Genotype-trait (GT) biplot indicating interrelationships among the measured traits from 35 sorghum genotypes.

A visualization of the relationship among the studied seven traits is facilitated by the G × T plot, an extension of GGE biplot where the traits are considered as testers (Figure 7). The association between any two traits is represented by the cosine of the angle between their vectors, as explained for the environments. The shorter vector lengths indicate a minimal variation among genotypes for the trait. The G × T biplot explained 47.1% of the total variation. The vector lengths in Figure 7 suggest that all the traits taken in this study have contributed to a variation. Based on the relationships among these traits, appropriate planning can be done in the breeding for yield and grain mold resistance. The important associations revealed by these biplots were: (i) GY has a strong positive association with flowering time, HGW, and PH, as indicated by the acute angles. (ii) Similarly, GMS had a positive association with PH and glume coverage as indicated by the acute angles between their vectors. Thus, indicating that taller lines and lines with more GC are resistant to grain mold. The right angle between GY and GMS indicates an independent relationship among these traits. The correlation coefficients worked out with the pooled data over environments also indicated the similar results (Table 7), supporting the results from the GT biplot with a positive association of GY with flowering time, HGW, and PH. GMS had a significant positive correlation with PH and glume coverage, and the association of GMS was observed to be non-significant with GY.

**Table 7:** Coefficient of correlation between the studied traits across environments during 2018.

**Trait**	**DF**	**PH**	**GC**	**PL**	**GY**	**HGW**	**GMS**
DF	1						
PH	−0.072	1					
GC	0.002	0.206	1				
PL	0.077	0.174	0.272	1			
GY	0.35^*^	0.312	0.028	−0.06	1		
HGW	−0.296	−0.02	−0.14	0.05	0.23	1	
GMS	−0.12	0.426^**^	0.430^**^	0.069	−0.042	−0.253	1

* *p < 0.05;*

** *p < 0.01; DF, Days to flower; PH, Plant height; GC, Glume cover; PL, Panicle length; GY, Grain yield; HGW, 100-grain weight; GMS, Grain mold score*.

**Figure 8:**
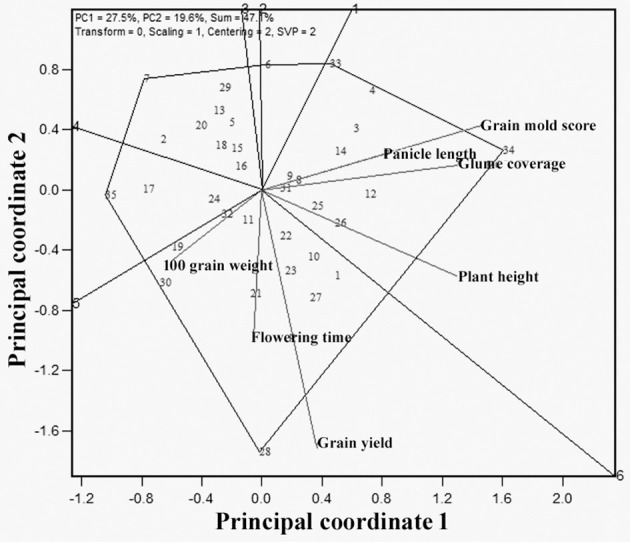
Polygon view of GT biplot indicating which is best for what among the 35 sorghum genotypes.

The “Which is best for what” analysis similar to which-won-where helped to identify the superior genotypes for particular trait(s) (Figure 8). The GT biplot shows that G34 was best for grain mold resistance, glume coverage, PH, and PL, whereas G28 was the best genotype for GY, flowering, and 100-seed weight. The genotypes that were found to have reasonably desirable grain mold score and more than average GYs were G8, G9, G31, G25, G26, and G12 from sector 1 and G10, G23, and G27 in sector 2.

## Discussion

Grain mold is the production constrain of rainy season sorghum resulting in the physical and chemical deterioration of the grain, which leads to the reduction in grain size and blackening of grain, and thus making them unfit for human consumption. The molded grains cannot be used for food due to reduced processing qualities. The quality of food grains affected with grain mold is drastically decreased, and thus improving the resistance to this disease, which is a major breeding concern. The use of host-plant resistance through the resistance cultivars is the most cost-effective and eco-friendly practice among the available recommended management strategies to control mold infestation (Mofokeng et al., 2017). Global research on sorghum grain mold emphasized on the identification of disease resistance-associated grain properties, such as GHR due to a more corneous percentage of endosperm, loose panicle, increased glume coverage, pigmented glume and testa, red pericarp, phenolic compounds, and flavan-4-ols (Esele et al., 1993, 1995; Bandyopadhyay et al., 2000; Aruna and Audilakshmi, 2004; Audilakshmi et al., 2005; Thakur et al., 2006; Sharma et al., 2010; Little et al., 2012; Thirumala Rao et al., 2012). Thus, the mechanisms governing grain mold resistance are quite numerous with each contributing meagerly and making the task of resistance breeding more difficult. Genotypic variations are available for each of these traits, but combining the desirable levels of these traits into one genotype is practically challenging. Successful resistance breeding program should be able to develop stable resistant lines with higher yields. Many breeding efforts were made to conquer the problem of grain mold by the pedigree breeding method using *zerazera/caudatum* line and many other grain mold-resistant sources (Ambekar et al., 2011; Prom et al., 2020). In the present study, 33 derivatives from the population breeding program are evaluated in the four testing locations. The differential ranking of genotypes across locations (hereafter referred to as environments) is caused due to a crossover GEI (Mohammadi and Amri, 2013), and a significant GEI for yield and grain mold in sorghum has been reported earlier (Rakshit et al., 2012; Aruna et al., 2016; Diatta et al., 2019).

### Performance of Genotypes and Heritability

For GY, the location component explained 51.7% of the total variation, while for grain mold score, the G × L component contributed to a higher proportion of variation. The greater influence of environment on sorghum phenology and grain traits, including grain mold, was reported earlier (Kenga et al., 2006; Gasura et al., 2015; Diatta et al., 2019). The h^2^bs of all the traits was generally observed to be high (≥0.50) but there was a slight difference in the individual location. Moderate to high h^2^b for DF and PH (Kenga et al., 2006; Jimmy et al., 2017) and for grain traits in different studies were reported (Almeida Filho et al., 2014; Mohammed et al., 2015; Belay and Meresa, 2017; Phuke et al., 2017). For grain mold resistance, Rodríguez-Herrera et al. (2007) and Diatta et al. (2019) reported high h^2^b, whereas Audilakshmi et al. (2011) reported low h^2^b (0.24–0.26). The high h^2^b found for most of the traits in this study indicates the additive gene action controlling these traits, which can be improved through selection (Kamatar et al., 2015).

### GGE Biplot Analysis for GY and Grain Mold Resistance

The GGE and GT biplots are useful for identifying ideal genotypes and test locations, deriving the relationships among traits, and detecting the best genotypes for different traits. In this study, the complexity of the GEI for GY and grain mold resistance was investigated using the GGE biplot analysis. Highly significant mean sum of squares (Table 2) indicated the significant variability among the genotypes, the prominence of variation due to locations, and a differential response of the genotypes to the environments for GY, grain mold resistance. More than 70% of the variability for most of the traits was explained by the first two PCs. The G + GL explained >10% of variability for GY and grain mold score indicates that the biplots can be used for further interpretation. For GY, the magnitude of genotype effects and GEI tested across four locations were smaller than that of environment main effect. It was observed that both crossover and non-crossover types of GEI were exhibited by the genotypes.

Information on genotype performance is also provided by the GGE biplot. Among the genotypes evaluated, G28 was the highest-yielding genotype followed by G21 (Figure 2). However, G28 was less stable when compared with other genotypes, whereas G21 expressed good stability across locations for GY. The performance of G28 perhaps suggest that a high-yielding genotype not necessarily a broad adaptation, hence it is suitable for specific adaptation. Otherwise, most stable ones are might not be high yielding (Rakshit et al., 2012; Bose et al., 2013; Aruna et al., 2016). For grain mold resistance, besides the resistant source B 58586 (G34), G4, and G33 were found to perform better and they also showed high stability for resistance.

The identification of an ideal genotype (one with high performance and greater stability) in the graphical presentation of GEI is an important application of the biplot (Yan and Tinker, 2006). From the graph, the genotype with the longest vector length and zero GEI, and located at the center of the concentric circles as in Figure 3 is taken as an ideal genotype. Genotypes located closer to the “ideal genotype” are more desirable than those placed far away. Thus, G28, G21, G27, and G10 were closer to an ideal genotype for GY, whereas G34, G4, G33 for grain mold resistance. It is difficult to visualize from the mean table. These genotypes can be used in the breeding program and further improved for the development of stable high GY lines with better pyramiding of grain mold resistance.

The highest-yielding genotype, G28 performed the best at Dharwad, above average performance at Hyderabad, and average performance at Akola and Parbhani. The best resistant line, G34 has given its best performance at Dharwad, and in all other places its performance was good and above average. The closer angles between the trait vectors represented a closer positive relationship. The study showed that Hyderabad, Dharwad, and Akola were positively correlated, whereas Parbhani had a negative association with the other three locations for GY indicating the existence of crossover GEI for GY. The ranking of genotypes has not changed much between Hyderabad, Dharwad, and Akola, but it changed in the Parbhani location. The genotypes showing a mixture of crossover and non-crossover types of GEI in MET data is commonly reported (Fan et al., 2007; Sabaghnia et al., 2008; Rao et al., 2011; Rakshit et al., 2012; Aruna et al., 2016) which is attributed to the genotypic response to varying environments such as cropping season, rainfall, temperature regime, RH, and light intensity apart from the geographical situation of locations (latitude, longitude, and altitude) (Saeed and Francis, 1984; Dehghani et al., 2006). For grain mold resistance, Dharwad, Parbhani, and Hyderabad were positively correlated, whereas Akola had no linear correlation with Dharwad and Parbhani. In both Dharwad and Hyderabad, the rainfall and humidity were higher during the harvest months of September and October in both the years 2017 and 2018 (Figure 1). Hence, the grain mold incidence was high at these two locations, which are the ideal locations for carrying out grain mold screening. This result implies that the resistant entry identified in Hyderabad may reflect similar *per se* performance in Dhaward and Parbhani and *vice versa*. These locations show a good discrimination among genotypes as well as represent other testing locations and serve as appropriate breeding and testing locations. The ideal locations are those that show a good discrimination among genotypes as well as represent other testing locations. These are the locations with good general adaptation and may reduce the cost of experimentation and also improves selection efficiency.

The “which-won-where” biplot graphically summarizes the crossover GE through creating different environmental clusters and identifying the genotypes with specific adaptation (Gauch and Zobel, 1997; Yan et al., 2000; Yan and Tinker, 2006; Putto et al., 2008; Rao et al., 2011; Rakshit et al., 2012; Aruna et al., 2016). A genotype suitable for one or more locations can be recommended for cultivation. Based on this analysis, the present testing locations were partitioned into three clusters for GY. Cluster 1 holds Dharwad and Akola with G28 as the best genotype. Cluster 2 is Hyderabad with G27 as the best genotype, and Cluster 3 is Parbhani with G13 as the best genotype. For grain mold score, the test locations were partitioned into two clusters. Cluster 1 had Dharwad and Parbhani with G34 as the best genotype and Cluster 2 had Hyderabad and Akola with G4 as the best genotype. Thus, partitioning the target environments into different clusters and placing different genotypes specific for different clusters is the best way to exploit the positive GEI (Yan and Tinker, 2005). Such studies are available in sorghum (Rao et al., 2011; Rakshit et al., 2012). The identification of such location clusters has an advantage in region-specific grain sorghum breeding selections and cultivation.

The information generated through the GT biplot can be used to aid genotype selection based on the specific traits and helps to decide on the trait index to be assigned to these traits in the selection process. A key indicative of the GT biplot is the interrelationships among different traits and a comparison of genotypes based on the multiple traits. The GT polygon also indicated that G34 is promising for grain mold resistance, glume coverage, PH, and PL, whereas G28 is promising for GY, flowering, and 100-seed weight. G8, G9, G31, G25, G26, G12, G23, and G27 were found to have a better grain mold tolerance and more than average GYs (Table 8). Most of the promising genotypes with low grain mold score were found to have colored glume, supporting the earlier studies (Nida et al., 2019). As the glume covers the grains, it may shield the grain from fungal invasion, which are later on supported by the chemical and physical properties of grain thus imparting resistance to grain mold (Stenhouse et al., 1997). GCL showed a strong association with mold resistance (Ghorade et al., 2014). Generally, darker glume provides better mold resistance. A negative correlation of grain mold incidence with GC (*r* = −0.56) was reported. It may be possible to enhance grain mold resistance in white-grained sorghum by incorporating a colored glume character. Panicle structure (loose, semi-compact, and compact) determines the microclimate around the seed during the postinfection of mold colonization. Loose panicle generally dries quicker after rain than a compact one and thus influence mold development. For instance, grain mold-resistant sources are usually having loose panicles. In this study, some genotypes were identified with resistance at par with the resistant sources besides the acceptable agronomic traits like semi-compact panicle, bold seed, and less GC, indicating that the population breeding approach helps in the accumulation of favorable alleles responsible for desirable traits into a designer genotype. This approach can also be used for essentially derived varieties for grain mold and GY traits.

**Table 8:** Some lines promising for both GY and grain mold.

**S. No**	**Genotype**	**Grain yield**	**Grain mold score**	**DF**	**PH**	**100 grain wt**	**Grain hardness**	**Glume cover**	**Glume color**	**Panicle compactness**
1	G28	71.58	4.17	80	191	2.69	7.17	51	Light red	SC
2	G21	61.29	4.57	76	152	2.36	9.33	70	White	SL
3	G23	55.63	3.83	77	174	2.36	7.33	57	Light brown	SL
4	G27	53.83	3.56	79	198	2.36	9.17	56	Light brown	SC
5	G8	52.35	3.40	70	166	2.59	9.83	50	White	SC
6	G25	49.88	3.61	77	180	3.61	9.17	45	Red	SC
7	G31	48.52	3.31	75	150	2.39	8.33	51	Brown	SL
8	G26	47.77	3.74	73	221	2.41	7.33	50	Red	SL
9	G12	46.96	3.47	73	223	2.24	9.67	44	Brown	Loose
10	G9	42.5	3.43	77	170	2.38	9.17	49	Red	SC
	B 58586	38.88	2.52	79	202	1.78	9.67	90	Light red	Loose
	296 B	42.75	7.75	79	134	2.35	7.83	36	White	SC
	lsd	9.84	0.90	3.1	8.08	0.24		7.45	-	-

*SC, Semi-compact; SL, Semi loose*.

The G × T plot indicated that GY and GMS are genetically independent of each other suggesting that an improvement for both GY and grain mold resistance can be achieved together. This is also in conformity with the idea that current practices of cultural/chemical disease control measures can improve the yield by controlling grain mold. Based on the trait associations, GY improvement could be achieved through PH, flowering time, and HGW using a correlated response to selection. Plant and GY component traits such as grain weight and 1,000-grain weight contributed toward higher GY (Tovignan et al., 2016; da Silva et al., 2017). Though a few earlier studies reported a negative correlation between grain mold resistance and yield components (Reddy et al., 2008; Diatta et al., 2019), they could find some genotypes that could combine good GY and grain mold tolerance, suggesting the possibility to break the negative linkage by growing and exercising selection in large segregating populations (Ambekar et al., 2011; Diatta et al., 2019). A previous study reported that climatic factors like high RH (85–100%) and temperature (25–30°C) prevail during crop maturity (September) favors the natural mold infestation (Tonapi et al., 2007). In such conditions, if there are some genotypes having low infestation, this could be attributed to their level of tolerance to mold infestation in comparison to other genotypes. Hence, in addition to genetic parameters, weather parameters in testing season are mandatory in grain mold resistance breeding nurseries and trials.

## Conclusions

The present study assessed the multilocation field performance of 33 derivatives of the population breeding approach, for their GY and grain mold resistance. The results showed a significant GEI on both GY and grain mold. The h^2^bs was generally high, indicating the possibility of improvement through a progeny selection for these traits. The GGE biplot analysis helped to identify G28 and G21 as the best candidates for GY, whereas G4, G33 were good for grain mold resistance in terms of their mean performance and stability compared to other lines. The study also identified the lines with high GY and moderately high grain mold resistance. The study further suggested that Dharwad is an ideal location for the evaluation of both GY and grain mold resistance. It is proved that population breeding, by involving diverse and elite parents, is effective in bringing together agronomically desirable characters thus help in improving grain quality in terms of grain mold resistance. Therefore, the demand for rainy season sorghum will increase for food, feed, and starch industries. Genetic material in this study also merits further genomic studies and marker development for mold tolerance. Hence, any improvement in grain quality cannot be separated from the improvement for grain mold resistance, which benefits the farmers with premium price in the markets.

## Data Availability Statement

The original contributions presented in the study are included in the article/supplementary material, further inquiries can be directed to the corresponding authors.

## Author Contributions

CA: conceptualization, data curation, writing—original draft, and manuscript preparation. ID: execution of the experiment at different centers and manuscript preparation. PR: analysis of the data, preparation of tables, graphs, and manuscript preparation. RG and VK: execution of the experiment at Akola and the collection of field data. AG: execution of the experiment at Akola and scoring of samples for grain mold resistance. SK and NH: execution of the experiment at Dharwad and the collection of field data. SC: execution of the experiment at Dharwad and scoring of samples for grain mold resistance. SM and KK: execution of the experiment at Parbhani and the collection of field data. VG: execution of the experiment at Parbhani and scoring of samples for grain mold resistance. CD and DB: field experimentation and data collection at Hyderabad. NK: recording data on GHR at Hyderabad. MG: data interpretation and manuscript preparation. VT: project administration and resources. All authors contributed to the article and approved the submitted version.

## Conflict of Interest

The authors declare that the research was conducted in the absence of any commercial or financial relationships that could be construed as a potential conflict of interest.

## Publisher's Note

All claims expressed in this article are solely those of the authors and do not necessarily represent those of their affiliated organizations, or those of the publisher, the editors and the reviewers. Any product that may be evaluated in this article, or claim that may be made by its manufacturer, is not guaranteed or endorsed by the publisher.
